# The Quest for Green Solvents for the Sustainable Production of Nanosheets of Two-Dimensional (2D) Materials, a Key Issue in the Roadmap for the Ecology Transition in the Flatland

**DOI:** 10.3390/molecules28031484

**Published:** 2023-02-03

**Authors:** Jessica Occhiuzzi, Grazia Giuseppina Politano, Gianluca D’Olimpio, Antonio Politano

**Affiliations:** 1 Department of Physical and Chemical Sciences, University of L’Aquila, Via Vetoio, 67100 L’Aquila, Italy; 2 Department of Information Engineering, Infrastructures and Sustainable Energy (DIIES), University “Mediterranea” of Reggio Calabria, Loc. Feo di Vito, 89122 Reggio Calabria, Italy

**Keywords:** Cyrene, Polarclean, Iris, green chemistry, 2D materials, liquid-phase exfoliation

## Abstract

The recent advent of two-dimensional (2D) materials has had a ground-breaking impact on science and technology. To exploit in technology their unique thickness-dependent physicochemical properties, the large-scale production of 2D materials is mandatory, but it represents an open challenge still due to various pitfalls and severe limitations including the toxicity of state-of-the-art solvents. Thus, liquid-phase exfoliation based on green and bioderived solvents represents an ideal methodology for massive production. This is particularly crucial for introducing 2D materials in technological applications such as the production of drinking water and agri-food industrial processes. Here, we assessed the production of 2D nanosheets (specifically, graphene, WS_2_, MoS_2_) with liquid-phase exfoliation assisted by eco-friendly solvents, with a comparative evaluation of green solvents in terms of the yield and, moreover, the aspect ratio, defectivity, and crystalline quality of the produced nanosheets. In particular, we focus on the most promising green solvents in terms of the yield and the crystalline quality of the produced nanosheets: Polarclean, Iris, and Cyrene, which were compared with acetone/water mixtures, isopropyl alcohol (IPA), triethanolamine (TEA), aqueous solutions of urea, and an ethanol/water mixture as well as two toxic solvents largely used for the production of 2D nanosheets: N-methyl-2-pyrrolidone (NMP) and N, N-dimethylformamide (DMF). Remarkably, the density of defects was particularly low in the liquid-phase exfoliation with Polarclean, as indicated by the Raman spectrum of graphene, with the I(D)/I(G) ratio below 0.1. Furthermore, Polarclean and Iris also enable ink-jet printing with functional inks of 2D materials based on green solvents due to their low dynamic viscosity at room temperature.

## 1. Introduction

2D materials represent a promising platform for technology [1,2,3,4,5,6,7] because of their unique physicochemical properties associated with the atomic thickness [8,9,10,11,12,13,14,15,16,17,18,19,20]. This implies enormous versatility in various fields, ranging from electrochemical energy storage devices, sensing, photonics, optoelectronics, and energy storage/production [21,22,23,24,25]. However, the industrial exploitation of 2D materials implies the need to achieve the sustainable large-scale production of 2D materials with high crystalline quality and unaltered electronic properties [26,27]. As of mid-2022, the global graphene and graphene oxide installed capacity undoubtedly exceeds 12,000 tonnes per year, but utilisation is low as the orders lag significantly behind.

Taking China’s graphene industry as a case-study example, with the production volume of 2000 tonnes per year of graphene powders and 3.5 million m^2^ per year of graphene films [28], its capability for economically scalable production is absolutely satisfactory. The quality of the graphene products, nevertheless, differs tremendously in practice, as it is extremely dependent on the graphene sources, manufacturing techniques, and fabrication condition monitoring.

Evident inconsistencies persist in the flake size and number of layers of commercially available graphene samples, along with considerable differences in the defect density and impurity content. These wide variations in quality have resulted in exaggerated reports on the applications of graphene and complaints about unrepeatable performance.

While mechanical exfoliation suffers from non-scalable processes with scarce reproducibility [29], chemical vapour deposition necessitates specific substrates allowing for epitaxial growth [30,31,32,33,34,35,36], with consequent complications related to the etching of 2D layers from the substrate [37], resulting in flakes with degraded crystalline quality with high amounts of defects and metallic impurities [38] and/or polymer residuals from the transfer process, altering the physicochemical properties of transferred flakes of 2D materials [39]. The removal of the substrate is also a challenging issue for the preparation of graphene by Si sublimation from the SiC substrate [40].

While the bottom–up synthesis is evidently inadequate for massive production, the top–down approach, implying exfoliation from parental bulk crystals, is in principle more suitable for scale up [41,42,43]. However, one should consider that mechanical exfoliation fails in reproducibility and scalability. On the other hand, liquid-phase exfoliation (LPE) [44,45] represents a viable technique able to overcome such difficulties. Definitely, LPE is suitable for scaling up by ensuring the massive production of highly crystalline 2D materials [46]. However, the quest of the most suitable solvent for the process remains an open issue to date. Currently, the most commonly used solvents for LPE are N-methyl-2-pyrrolidone (NMP) and N, N-dimethylformamide (DMF), due to the strong compatibility of their values of surface tension and of the Hansen solubility parameters (HSP) with surface energy and HSP for graphite and other 2D materials (Table 1). Unfortunately, NMP and DMF have recently been listed as extremely hazardous substances for toxicity issues, with subsequent restrictions on their use in both Europe and the United States of America [46,47,48]. Accordingly, the scientific community is engaged in a quest for environmentally friendly solvents that are capable of replacing NMP and DMF for the LPE of 2D materials, with appropriate effectiveness.

## 2. Materials and Methods

*Materials:* WS_2_ (CAS number 12138-09-9), MoS_2_ (CAS number 1317-33-5), and graphite (CAS number 7782-42-5) were purchased from Sigma-Aldrich. Absolute ethanol and N-methyl-2-pyrrolidone (NMP) were purchased from commercial chemical suppliers.

Methyl-5-(dimethylamino)-2-methyl-5-oxopentanoate (Rhodiasolv Polarclean) and dimethyl 2-methylglutarate (Rhodiasolv Iris) were provided by Rhodiasolv, Solvay Novecare, Paris.

*Exfoliation:* The preparation of the methodology for the exfoliation of the layered materials was performed by considering 0.05 g of the powders (WS_2_, MoS_2_, and graphite) dispersed in 40 mL of the solvents under investigation (NMP, Rhodiasolv Polarclean, Rhodiasolv Iris).

The solution was sonicated for 4 h in a sonicator bath (Labsonic LBD2 working at 40 kHz) with a thermostat built into it to prevent excessive temperature rise (set not to exceed 25 °C). Next, it is necessary to completely remove the solvent used from the solution. This mechanism is accomplished by numerous centrifugations.

The sequence of centrifugations started with an initial centrifugation at 5000 rpm for 20 min, at the end of which the supernatant was discarded and replaced with the same amount of ethanol. The second step saw further centrifugations aimed at removing the residue of the solvent used, with a final centrifugation at 1000 rpm.

This last centrifugation was performed to try to separate the thinner flakes from the thicker part of the material that had not been exfoliated. At the end of this process, the supernatant from the last step was taken for later characterisation.

## 3. Results and Discussion

Among the ideal characteristics that the solvent must have, it is important to consider their values of polarity, surface tension, viscosity, and toxicity [49]. In particular, a suitable solvent for LPE should minimize the energy input required to overcome the van der Waals forces for effective sheet separation [50]. This corresponds to the minimisation of the enthalpy of mixing per unit of volume (Δ*H*/*V*). In turn, it is related to the Helmholtz energy of solvent (*F_solv_*), the thickness of the flakes (*T_layered_*), the free Helmholtz energy of layered materials (*F_layered_*), and the volume fraction (*φ*) through [51,52]:

(1) $$\frac{\Delta H}{V}\sim \;\frac{2}{T_{layered}}{\left(\sqrt{F_{solv}}-\sqrt{F_{layered}}\right)}^{2}\phi \;$$

with

(2) $$F_{layered}=(\sigma_{s}-{\mathrm{TS}}_{\mathrm{Sur}})$$

where $\sigma_{s}$ is the surface energy and S_Sur_ is the surface entropy.

Therefore, matching the surface tensions of the solvent (Table 2) and layered materials (Table 1) is crucial to achieve an efficient LPE. However, another critical issue is related to the dispersibility of the flakes and solvent, which depends on the specific molecular interactions between the solvent and the solute. The evaluation of the dispersibility of both nanosheets and the solvent can be carried out based on the assessment of HSP (Table 2), which describes the interaction between the solvent and the solute. Precisely, HSP considers the dispersion forces (δ_d_), polarity interactions (δ_p_), and hydrogen bonds (δ_h_), respectively. If the HSP of solvents has comparable values with the solute, the energy cost for their dispersion is minimised.

**Table 1:** Surface energy and Hansen solubility parameters for graphite, MoS_2_, and WS_2_.

	Surface Energy E_sur_ [mNm^−1^]	Hansen Solubility Parameters		
		δ_d_ [MPa^1/2^]	δ_p_ [MPa^1/2^]	δ_H_ [MPa^1/2^]
Graphite [47]	≈62	≈18	≈9.3	≈7.7
MoS_2_ [53]	≈70	17–19	6–12	4.5–8.5
WS_2_ [53]	≈75	16–18	5–14	2–19

Volatile organic compounds (VOCs such as isopropyl alcohol and ethanol), although they appear as good alternatives to NMP and DMF, unfortunately, they suffer from insufficient exfoliation yields, which are inevitably halved [54] due to the need to transfer nanosheets from a suspension into NMP [55]. Moreover, their flash temperature is often around 12–13 °C, which could result in being hazardous for industry.

With regard to the possibility of using surfactants in aqueous media [56], most of them are insulating and their residuals [57] are thus detrimental in many applications requiring thermal and electrical conductivity.

Electrochemical exfoliation (both anodic and cathodic) in aqueous electrolytes has emerged as a novel platform for the production of 2D materials [58]. However, for bulk semiconductors or insulators, electrochemical exfoliation is ineffective in breaking the interlayer van der Waals forces without including a conducting additive [59]. Furthermore, reaching the monolayer regime through the electrochemical exfoliation of bulk materials remains a severe hurdle [60]. Another problem is related to the unconventional operational electrochemical conditions, which imply the occurrence of oxygen and hydrogen evolution stimulated by electrochemical polarisation [61]. Finally, electrochemical exfoliation in aqueous electrolytes typically afford flakes of 2D materials with a high number of defects [58,62].

Recently, TEA [63] and urea aqueous solutions [64] have been proposed as green alternative media for the LPE of graphene and other layered materials. Regarding TEA, though it shows good results in terms of the flakes’ microstructure and dispersion stability, issues related to the yield of the process, and mainly to the chemical modification of flakes induced by possible functionalisation [65,66] during the process are still open. In addition, the very high dynamic viscosity (605.9 cP at T = 25 °C [67]) precludes the use of such dispersions for the inkjet printing of 2D material-based inks, for which the viscosity range is recommended to be 1–10 cP [68]. On the other hand, aqueous dispersions of urea have shown encouraging results for graphite exfoliation, obtaining high quality flakes. Nevertheless, the low yield of the process (2.4%), evidently related to the significant difference in the surface energy (Table 2), makes urea inappropriate for scalability.

**Figure 1:**
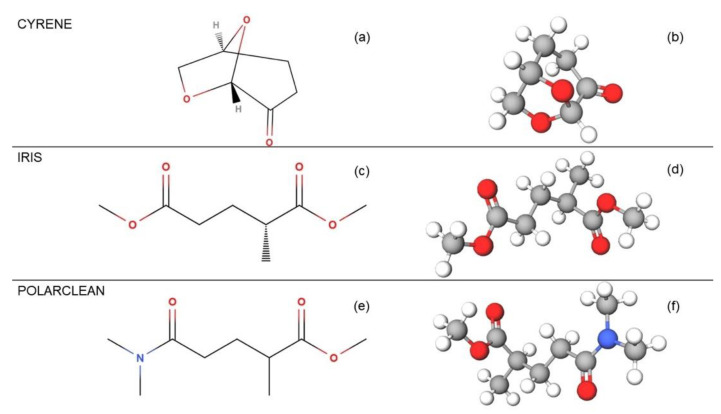
Comparison of the molecular structures of the solvents used: Cyrene, Iris, and Polarclean, whose atomic structures are shown in (**a**,**c**,**e**) plain and (**b**,**d**,**f**) ball-and-stick layouts, respectively.

Among the various attempts in the literature, the most effective green solvents for the LPE of 2D materials appear to be: (i) dihydrolevoglucosenone (Cyrene, CAS: 53716-82-8) [69]; (ii) methyl-5-(dimethylamino)-2-methyl-5-oxopentanoate (Rhodiasolv Polarclean, CAS:1174627-68-9) [70]; and (iii) dimethyl 2-methylglutarate (Rhodiasolv Iris, CAS: 33514-22-6) [71] (Figure 1).

Cyrene (C_6_H_8_O_3_) has been considered in recent studies [69] to be the most viable substituent to NMP as an organic solvent. It does not exhibit the amide functionality associated with the reproductive toxicity of many of the common dipolar aprotic solvents. This solvent limits the production of corrosive or polluting by-products at the end of its cycle due to the lack of chlorine. In addition, unlike other petrochemical dipolar aprotic solvents, which, on decomposition, tend to release NO_x_, Cyrene has a flash point of 108 °C, it is stable, and after biodegradation, it releases only carbon dioxide and water. However, it should be mentioned that Cyrene has an acute toxicity (LD50 > 2000 mg/kg) and aquatic toxicity (EC50 > 100 mg/L), which makes its use for drinking water production impossible. Moreover, its use in inkjet printing is also impossible because of the high dynamic viscosity (14.5 cP at T = 20 °C).

Polarclean (C_9_H_17_NO_3_) could represent a more effective candidate for the massive production of 2D materials by LPE, based on results in [70]. It does not show toxicity up to 1000 mg/ (kg day), it is biodegradable, and non-mutagenic, thus being safer than oxygenated solvents such as VOCs. The water solubility of Polarclean is more than 490 g/L under room temperature conditions (25 °C) and it has a melting point at an ambient pressure of 160 °C. Currently, Polarclean is mostly used for the solubilisation of agrochemicals as well as for crop protection and animal nutrition [72]. Recently, the use of Polarclean has been extended to the production of polymeric membranes for ultrafiltration and water desalination for the production of drinking water [73], the synthesis of bio-based aliphatic polyurethanes [74], the dimerisation of abietic acid [75], and for copper-catalysed azide–alkyne cycloaddition [76].

Polarclean is absolutely compatible for use in drinking water production or for the agrifood industry. Moreover, its dynamic viscosity of 9.78 cP (at T = 23 °C) makes it suitable for ink-jet printing, contrary to Cyrene.

Another promising eco-friendly solvent could be Iris (C_8_H_14_O_4_), considering the very recent findings [71]. Iris has an excellent safety profile: it is nontoxic, biodegradable, non-carcinogenic, and non-irritating. This solvent has the lowest of the toxicity levels investigated; in fact, its dosage can be as high as 2000 mg/(kg day) without any detectable toxicity. The enormous potential of this solvent also lies in its flash point being as low as 90.8 °C, which reduces the flammability risks, and moreover, facilitates the removal of the solvent by evaporation. Its solubility in water is greater than 25 g/L at a temperature of 23 °C. With a dynamic viscosity value as low as 2.85 cP at 20 °C, it suitable for use in inkjet printing, like for Polarclean.

Table 3 reports the density, the boiling point, and the dynamic viscosity at room temperature of the various solvents used for LPE.

**Figure 2:**
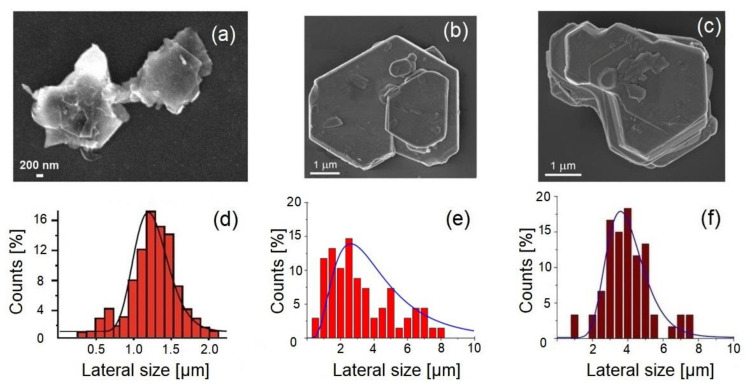
Comparison of the representative high-resolution SEM images and statistical analysis determined from the images. Exfoliated nanolayers with the LPE of WS_2_, assisted by (**a**) Iris, (**b**) Polarclean (taken from [70] with permission), (**c**) NMP. Lateral scale size distribution analysis of WS_2_ with (**d**) Iris, (**e**) Polarclean (taken from [70] with permission), (**f**) NMP.

To assess the quality of the nanosheets of the 2D materials produced by LPE with a specific solvent, it is straightforward to evaluate the lateral size (checked by electron microscopies such as scanning electron microscopy (SEM) and transmission electron microscopy, TEM) and thickness (checked by atomic force microscopy, AFM) of the nanosheets of the same set of layered materials: graphite, MoS_2_, and WS_2_. Figure 2 shows the microscopical images and statistical analysis to assess the lateral size of flakes produced with LPE assisted by Polarclean and Iris. Remarkably, NMP-assisted LPE resulted in the formation of flakes with sharp edges and defined angles (similarly to the case of Polarclean), with an average lateral size of around 3–4 nm, which emerged from the analysis of the distribution (Figure 2f). However, the thickness analysis by AFM measurements revealed the incomplete exfoliation. Definitely, when comparing the collected microscopical images, it was evident that the flakes exfoliated with Polarclean (in the case of WS_2_) and Iris (in the case of MoS_2_) were thinner (see the AFM experiments in [70,71]) than those obtained with NMP with the same experimental procedure.

**Figure 3:**
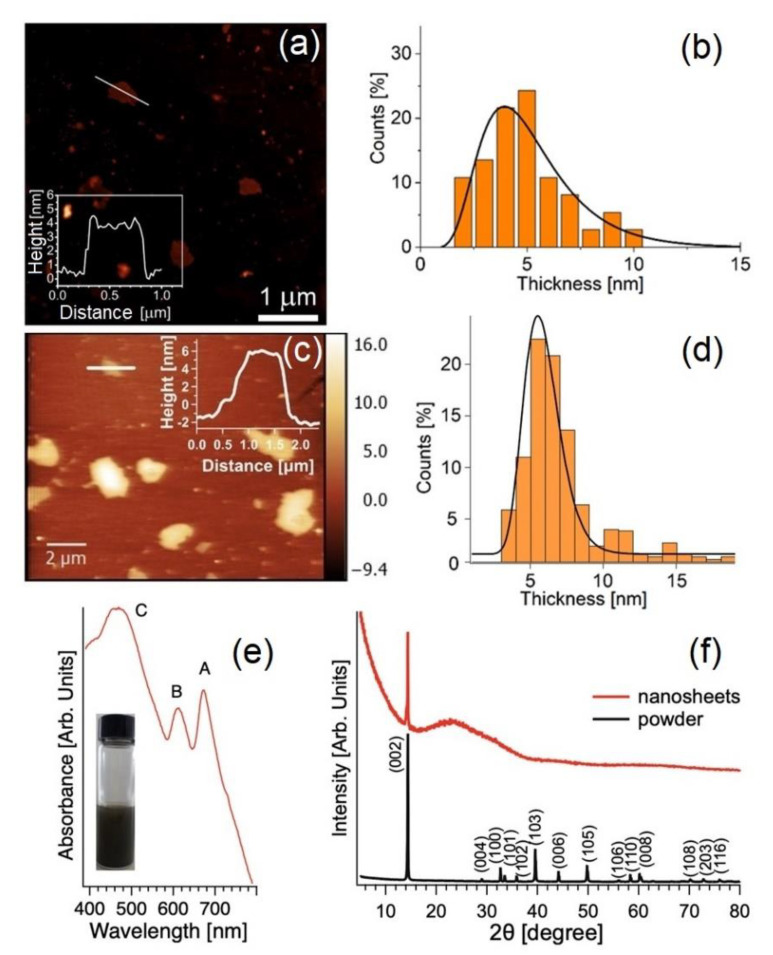
Representative AFM image of MoS_2_ flakes in (**a**) Polarclean (taken from [70] with permission) and (**c**) Iris. The height profile along the white solid line is reported in the inset. Analysis of the thickness distribution (**b**,**d**) was determined from the AFM measurements. (**e**) UV–VIS spectrum of the MoS_2_ nanosheets produced by Iris-assisted LPE. (**f**) XRD pattern of the (black curve) powdered MoS_2_ bulk crystals and (red curve) exfoliated nanosheets of MoS_2_.

Remarkably, the use of these innovative green solvents did not alter the electronic properties of the 2D materials, as inferred from the UV–VIS absorption spectra in Figure 3 (exhibiting excitons for both MoS_2_ and WS_2_, being 2D semiconductors [70,71]). Repeating the UV–VIS spectra in a timescale of months also enabled us to secure the stability of the produced functional inks.

Figure 3a–d reports the representative AFM measurement and statistical thickness distribution for the case study example of WS_2_ produced by LPE with Polarclean and Iris. In both cases, the average thickness was around 5 nm, thus confirming the efficient exfoliation in atomically thin layers.

X-ray diffraction (XRD) is the most reliable technique to check the integrity of the atomic structure after the breakage of van der Waals bonds. Figure 3f reports the crystal structure of MoS_2_ nanosheets obtained by Iris-assisted LPE with respect to the bulk MoS_2_.

The appearance of the (002) peak at 14.4° in the XRD pattern of exfoliated MoS_2_ nanosheets secured their good crystallinity, congruent with the hexagonal structure of the bulk crystal, with peaks matching ICDD ref no. 04-003-3374.

The performances of Polarclean as an exfoliation medium for 2D materials was directly compared with the case of the most diffuse state-of-the-art solvent (i.e., NMP). Therefore, we also performed LPE under the same operating conditions for NMP (see Methods for the experimental procedures). While the lateral size was comparable, the statistical analysis on thickness revealed a bimodal distribution for 2D materials produced by NMP-assisted LPE, which peaked around 4 and 30 nm, corresponding to thin and thick flakes, respectively. Remarkably, ~85% of flakes exfoliated by Polarclean had a thickness <5 nm. One can deduce the prevalence of ultrathin flakes (1–3 layers) in Polarclean-assisted LPE. In contrast, the use of NMP in the same processing conditions produced flakes with an ~76% of thickness >5 nm, thus evidencing a largely incomplete exfoliation of the bulk crystal in NMP-assisted LPE.

**Figure 4:**
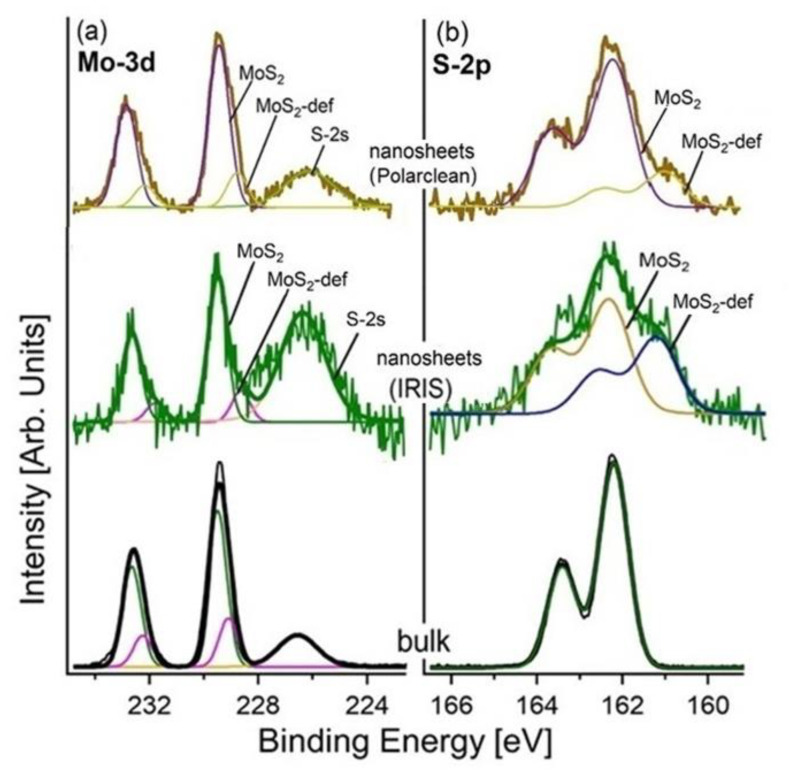
(**a**) The Mo-3d and (**b**) S-2p core levels of the bulk MoS_2_ and MoS_2_ nanosheets produced by LPE with Polarclean (taken from [70] with permission) and Iris. The component at a BE of 226.7 eV in the Mo-3d core level (in panel a) was related to the overlap of the S-2s core level. The photon energy was 1486.6 eV (Al Kα), and the spectra were normalised to the maximum.

More insights on the quality of the nanosheets produced by LPE with Polarclean and Iris were provided by X-ray photoelectron spectroscopy (XPS), as illustrated in Figure 3 for the case-study example of MoS_2_. The core-level spectra of the bulk and exfoliated MoS_2_ are shown in Figure 4. The Mo-3d core levels are split into J = 5/2 and 3/2 components shifted by 3.1 eV. Specifically, the Mo-3d core levels had two different contributions from pristine (fully coordinated atoms) and defective MoS_2_ (with sulphur vacancies), with a binding energy (BE) of 229.8 and 229.2 eV for the J = 5/2 component, respectively. Moreover, a minority component located at lower BE was associated with the presence of the defects due to a redistribution of the charge. Explicitly, the charge localised on the more electronegative sulphur atom, once it is desorbed, is redistributed on the first neighbouring atoms to increase the Coulomb screening effect [77,78]. Particularly, one could note in the Mo-3d spectra the lack of MoO_3_-derived spectral components, which should be present at a BE of 232.4 eV for the J = 5/2 component [79]. Therefore, one can infer that both Polarclean and Iris do not act as oxidation agents for MoS_2_ nanosheets, and congruently, Polarclean/Iris-assisted LPE process do not favour the oxidation of MoS_2_ flakes. Concerning the S-2p core levels, they were split in J = 1/2 and 3/2 components shifted by 1.2 eV. Two well-distinct contributions associated with pristine and defective MoS_2_ were observed at a BE of 162.5 and 161.5 eV for the J = 3/2 component, respectively, as in previous reports [80,81,82]. No trace of the sulphur-oxide phases was found, in contrast to the case of WS_2_, for which spectral contributions from both SO_4_ and SO_3_ exist.

A direct comparison between the XPS spectra of the Iris and Polarclean LPE-MoS_2_ only showed a higher presence of defects in the case of the MoS_2_ exfoliated with Iris.

**Figure 5:**
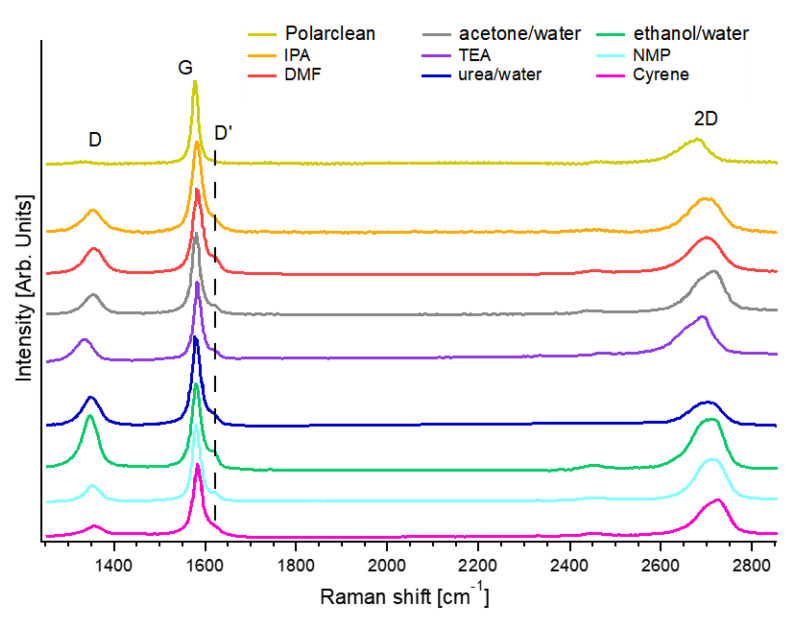
Comparison of the Raman spectrum for graphene exfoliated in the liquid phase with NMP [69], Cyrene [69], IPA [87], DMF [88], acetone/water [89], ethanol/water [90], TEA [63], and aqueous solution of urea [64], and Polarclean [70]. Data were taken from the above-mentioned references.

Concerning the exfoliation of graphene flakes with Polarclean, amazingly, the distribution of the lateral size reached an average value as high as 10 µm, absolutely one of the largest reported to date for the LPE of nanosheets starting from bulk graphite [12,19]. The related Raman spectrum (Figure 5) exhibited D and G bands at 1331 and 1581 cm^−1^, respectively. One should consider that whereas the G peak resulted from the E_2g_ optical phonon of graphene [83], the D band is produced by breathing modes of six-atom rings and necessitates a defect for its activation [84]. Consequently, the I(D)/I(G) ratio is a generally accepted probe of structural defects in the graphene layer [85]. Outstandingly, in the case of the Polarclean-assisted LPE of graphene, the I(D)/I(G) was 0.07 ± 0.01. Therefore, one can guess a density of defects of only (8 ± 2) × 10^9^ cm^−2^, congruently with the exceptional crystalline order of exfoliated graphene flakes (without indication of defects) in the HR-TEM images in [70]. For the cases of other solvents, the density of defects for graphene exfoliated by LPE was (6 ± 2) × 10^10^, (5 ± 2) × 10^10^, (1.0 ± 0.3) × 10^11^, (9 ± 3) × 10^10^, (4 ± 1) × 10^10^, (2.6 ± 0.7) × 10^11^, (6 ± 2) × 10^10^, (7 ± 2) × 10^10^ defects × cm^−2^ with NMP [69,86], Cyrene [69], IPA [87], DMF [88], acetone/water [89], ethanol/water [90], TEA [63], and aqueous solution of urea [64], respectively. Evidently, graphene flakes exfoliated with Polarclean displayed a density of defects lower by about one order of magnitude compared to LPE assisted by other solvents.

The inspection of the intensity of the D′ band could offer further clues on the density of defects. Comparable to the D band, the D′ mode is a double resonance due to the transverse optical (TO) phonons at K or K′, activated by defects, with the difference that it involves an intravalley rather than intervalley process [85]. Notably, the intensity of the D′ band at 1615 cm^−1^ was suppressed for the case of the Polarclean-assisted LPE of graphene, in contrast with the case of other solution processing methods (Figure 5).

## 4. Conclusions

Here, we assessed the choice of solvents for LPE for the production of atomically thin layers of van der Waals crystals. The most competitive solvents, both in terms of environmental sustainability and operability, were examined and compared with those that are more widely used, but unfortunately resulted in being harmful to human health. The most promising eco-friendly solvents in terms of the yield and crystalline quality of the produced nanosheets are Polarclean, Iris, and Cyrene. Among these solvents, one can note that the density of defects is exceptionally low in Polarclean-assisted LPE, as inferred by the Raman spectroscopy in graphene, with the I(D)/I(G) ratio of only 0.07. Moreover, Polarclean and Iris also enable ink-jet printing with functional inks of 2D materials based on green solvents.

The superior performances in LPE, together with the absence of any toxicity issue and its biodegradability, make green solvents such as Polarclean and Iris ideal candidates for the sustainable large-scale production of 2D materials. Naturally, they can also replace solvents commonly employed for other processing methods beyond sonication such as shear mixing [91] or wet-jet mill [92], particularly promising for industrial scale up. The efficiency of the green LPE process is crucial in order to combine intrinsic benefits for environmental health and safety with the optimisation of the performance. Undeniably, the introduction of a green solvent for LPE will also expand the growing market of 2D materials towards fields nearly unexplored (e.g., recovery of minerals from seawater, concentration of fruit juices, production of drinking water, etc.) to date, as a result of the toxicity of the state-of-the-art solvents for LPE, with subsequent superb impact on the commercial potential of their technological applications.

## Figures and Tables

**Table 2:** Surface tension and the Hansen solubility parameters for NMP, DMF, IPA, Cyrene, Polarclean, and Iris.

	Surface Tension σ_s_ [mNm^−1^]	Hansen Solubility Parameters		
		δ_d_ [MPa^1/2^]	δ_p_ [MPa^1/2^]	δ_H_ [MPa^1/2^]
NMP	40.1	18.0	12.3	7.2
DMF	37.1	17.4	13.7	11.3
IPA	21.7	15.8	6.1	16.4
Acetone	58.1	15.5	10.4	7.0
Ethanol	46.1	15.8	8.8	19.4
Urea 30% in H_2_O	74.0	17.0	16.7	38.0
TEA	45.9	17.3	7.6	21.0
Cyrene	72.5	18.7	10.5	6.9
Polarclean	38.0	15.8	10.7	9.2
Iris	33.0	16.6	8.7	5.0

**Table 3:** Density, boiling point, and dynamic viscosity at 25 °C for NMP, DMF, IPA, acetone, ethanol, aqueous urea solutions, Cyrene, Polarclean, and Iris.

Solvent	Density [g/cm^3^]	Boiling Point [°C]	Dynamic Viscosity at 20 °C [cP]
NMP	1.03	202	1.66
DMF	0.94	153	0.92
IPA	0.78	82	2.01
Acetone	0.78	56	0.32
Ethanol	0.79	78	1.09
Urea 30% in H_2_O	1.32	135	1.40
TEA	1.13	335	404
Cyrene	1.25	226	14.5
Polarclean	1.04	280	9.78
Iris	1.05	222	2.85

## Data Availability

Not applicable.
